# Incidence, Outcome, and Predictors of Intracranial Hemorrhage in Adult Patients on Extracorporeal Membrane Oxygenation: A Systematic and Narrative Review

**DOI:** 10.3389/fneur.2018.00548

**Published:** 2018-07-06

**Authors:** Alexander Fletcher-Sandersjöö, Eric Peter Thelin, Jiri Bartek, Mikael Broman, Marko Sallisalmi, Adrian Elmi-Terander, Bo-Michael Bellander

**Affiliations:** ^1^Department of Neurosurgery, Karolinska University Hospital, Stockholm, Sweden; ^2^Department of Clinical Neuroscience, Karolinska Institutet, Stockholm, Sweden; ^3^Division of Neurosurgery, Department of Clinical Neurosciences, University of Cambridge, Cambridge, United Kingdom; ^4^Department of Neurosurgery, Copenhagen University Hospital Rigshospitalet, Copenhagen, Denmark; ^5^Department of Medicine, Karolinska Institutet, Stockholm, Sweden; ^6^ECMO Center Karolinska, Karolinska University Hospital, Stockholm, Sweden; ^7^Department of Physiology and Pharmacology, Karolinska Institutet, Stockholm, Sweden

**Keywords:** intracranial hemorrhage, intracerebral hemorrhage, brain injury, neurological injury, extracorporeal membrane oxygenation, extracorporeal life support, adults

## Abstract

**Background:** Intracranial hemorrhage (ICH) is a common complication in adults treated with extracorporeal membrane oxygenation (ECMO).

**Objectives:** The aim of this study was to conduct a systematic review of the literature on the incidence, outcome and predictors of ECMO-associated ICH in adult patients, supplemented by a narrative review of its pathophysiology, management and future perspectives.

**Methods:** MEDLINE, EMBASE, Cochrane Database of Systematic Reviews and www.clinicaltrials.gov were systematically searched. Studies that reported incidence, outcome or predictors of ECMO-associated ICH in adults (≥18 years) were eligible for inclusion.

**Results:** Twenty five articles were included in the systematic review. The incidence of ECMO-associated ICH varied between 1.8 and 21 %. Mortality rates in ICH-cohorts varied between 32 and 100 %, with a relative risk of mortality of 1.27–4.43 compared to non-ICH cohorts. An increased risk of ICH was associated with ECMO-duration, antithrombotic therapy, altered intrinsic coagulation, renal failure, need of blood products, rapid hypercapnia at ECMO initiation, and even pre-ECMO morbidity.

**Conclusions:** ICH is a common complication in adults treated with ECMO and associated with increased mortality. Treating an ICH during ECMO represents a balance between pro- and anticoagulatory demands. Neurosurgical treatment is associated with severe morbidity, but has been successful in selected cases. Future studies should aim at investigating the validity and feasibility of non-invasive monitoring in early detection of ECMO-associated ICH.

## Background

### Rationale

Extracorporeal membrane oxygenation (ECMO) is being used more frequently in adults (1–5), and is now recognized as an important part in the treatment of severe reversible refractory respiratory and/or circulatory failure (1–3). However, the treatment itself is associated with significant morbidity and mortality (6), and intracranial hemorrhage (ICH) is one of the most frequent serious adverse events occurring during ECMO support (7–9). In fact, during the H1N1 pandemic in Australia and New Zealand, ICH was the most common cause of death among ECMO treated patients (10). Despite this, there are no established guidelines on its detection, prevention or management (11).

### Objectives

The aim of this study was to review the literature on ICH in ECMO-treated adult patients. This was performed by conducting a systematic review of the literature on the incidence, outcome, and predictors of ECMO-associated ICH in adults, supplemented by a narrative review of its pathophysiology, management, and future perspectives.

## Methods

### Search strategy and selection criteria

This review was performed by searching the following databases from their dates of inception until January 2017: MEDLINE, EMBASE, Cochrane Database of Systematic Reviews, and the clinical trial registry www.clinicaltrials.gov. A search strategy for MEDLINE and EMBASE was decided on (Supplementary File 1), with a similar search strategy utilized for the other databases. There was no specific restriction on study methodological quality. The titles and abstracts were independently screened to determine if they met the inclusion criteria. Full texts of the chosen articles were assessed to confirm this. Reference lists of relevant articles were screened for additional studies.

### Inclusion and exclusion criteria

All studies that reported incidence, outcome or predictors of ECMO-associated ICH in adults (≥18 years) were included. ICH was defined as an intraparenchymal hemorrhage (IPH), subdural hemorrhage (SDH), and/or subarachnoid hemorrhage (SAH). Studies were excluded if they were non-English or if it was impossible to deduce the data specifically related to ICH in ECMO-treated adults—for example if they failed to specify patient age, or had grouped ICH and other neurological complications (e.g., ischemic stroke) together and analyzed these as one entity.

### Data abstraction

Using a customized form, data were extracted from the included articles and stored in an electronic database. Where applicable, the following data were abstracted: study design, study length, amount of patients included, percentage of patients treated with V-A ECMO, amount of ICH cases, ICH characteristics, outcome studied, mortality rate, and risk factors for ICH development.

### Data analysis

A systematic analysis was performed comparing mortality rates in ICH vs. non-ICH adult ECMO cohorts, with data reported following the Preferred Reporting Items for Systematic Reviews and Meta-Analyses (PRISMA) (12) (Supplementary File 2). We presented the binary data as risk ratios with 95% confidence intervals and *p*-values. Additional systematic analyses, including meta-analysis, were not performed due to the heterogeneity of data and study design of the included articles. The raw data supporting the conclusions of this manuscript will be made available by the authors, without undue reservation, to any qualified researcher.

## Results

### Study selection and characteristics

**Figure 1:**
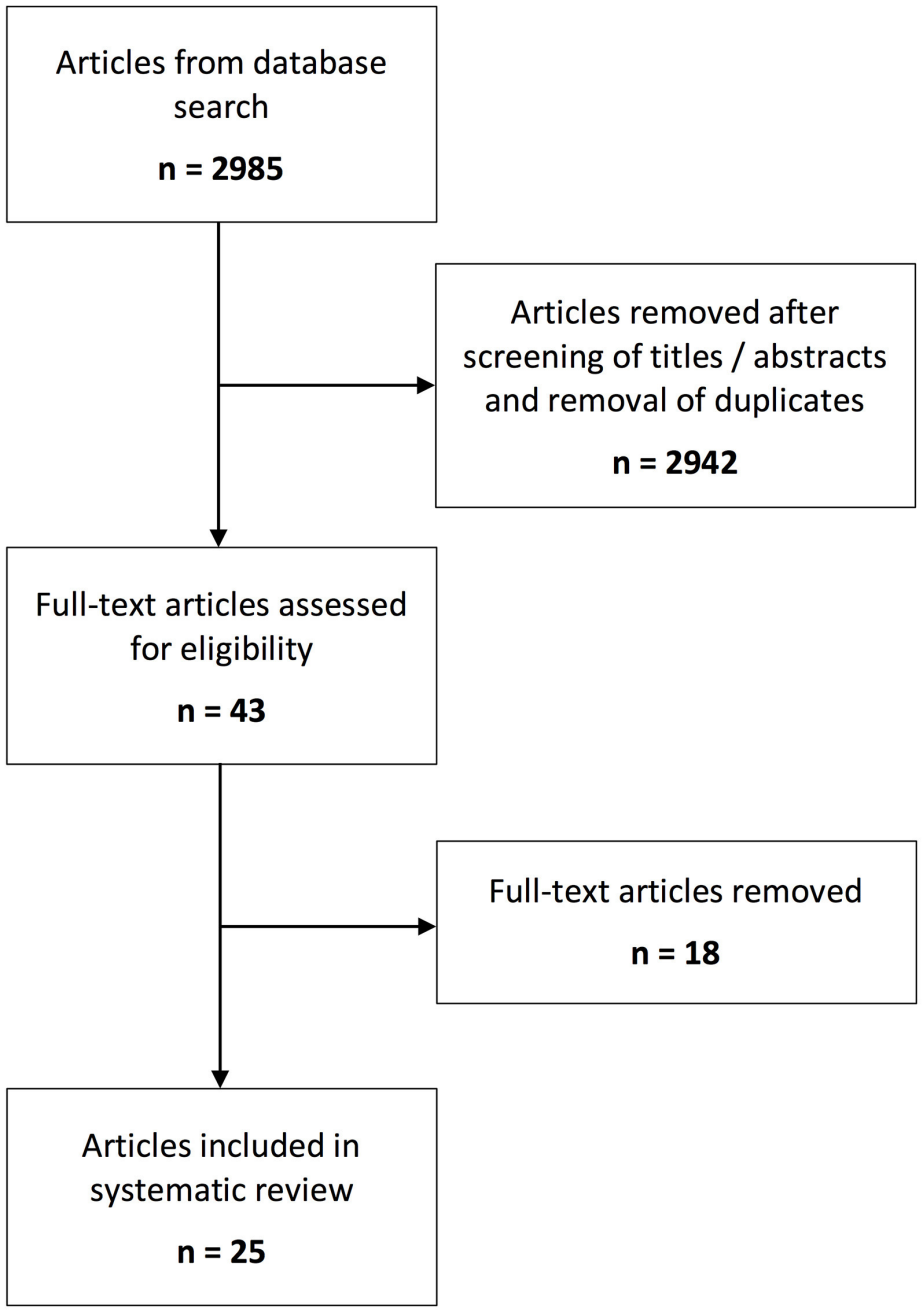
Schematic overview of the number of identified records for the systematic steps of the review process.

The initial literature search yielded 2,985 articles. 2,942 articles were excluded following removal of duplicates and title and abstract review. Following full-text review of the remaining 43 articles, 25 were included in the systematic analysis (Figure 1).

### Synthesized findings

The reported incidence of ICH in adults during ECMO varied between 1.8 and 21% (4, 5, 13–22) (Table 1). The vast majority of studies were retrospective, and only one prospective cohort study was included.

**Table 1:** Studies reporting ICH rates in adult ECMO cohorts.

**Study**	**Study design**	**Study length (years)**	**Patients (*n*)**	**% V-A ECMO**	**ICH (*n*)**	**ICH rate (%)**	**ICH characteristics**
Fletcher-Sandersjöö et al. 23	Single-center, retrospective	10.8	253	36	54	21.3	76% intraparenchymal hemorrhage (IPH), 2% subdural hemorrhage (SDH), 22% subarachnoid hemorrhage (SAH)
Kasirajan et al. 14	Single-center, retrospective	5.0	74	100	14	18.9	ICH was defined as IPH and/or intraventricular hemorrhage
Klinzing et al. 15	Single-center, retrospective	6.0	74	27	8	10.8	25% >1 type, 88% IPH, 38% SAH
Lockie et al. 16	Single-center, retrospective	4.2	250	0	41	16.4	37% > 1 type, 20% large IPH, 39% petechial IPH, 56% SAH, 2% SDH
Lorusso et al. 18	Multicenter, ELSO data registry	22.0	4,522	100	80	1.8	NR
Lorusso et al. 17	Multicenter, ELSO data registry	24.0	4,988	0	181	3.6	NR
Luyt et al. 19	Single-center, retrospective	7.0	135	0	10	7.5	NR
Nasr and Rabinstein 4	Multicenter, Nationwide inpatient sample	11.0	8,397	NR	239	2.9	NR
Omar et al. 20	Single-center, retrospective	7.0	154	81	12	7.8	NR
Paden et al. 21	Multicenter, ELSO data registry	NR	NR	NR	NR	3.9	NR
Rastan et al. 24	Single-center, prospective	12.0	517	100	19	3.7	NR
Smedira et al. 22	Single-center, retrospective	7.5	202	100	13	6.4	NR

ICH, intracranial hemorrhage; IPH, intraparenchymal hemorrhage; SDH, subdural hemorrhage; SAH, subarachnoid hemorrhage; ECMO, extracorporeal membrane oxygenation; ELSO, Extracorporeal Life Support Organization; V-A, venoarterial; NR, not reported

Survival rates in adult ECMO patients who developed ICH was low, with a reported mortality rate of 32–100% (7, 10, 14–17, 19, 20, 23, 25). A systematic analysis comparing outcome between ICH and non-ICH cohorts is presented in Table 2. Of these, the two largest studies (*n* > 200) reported a 2.91 and 1.48 relative risk of mortality comparing their ICH and non-ICH cohort (13, 16), while the two studies including the smallest cohorts reported the highest relative risk [4.43 and 3.28, respectively] (10, 25) (Table 2).

**Table 2:** Systematic analysis of mortality rates between ICH and non-ICH adult ECMO cohorts.

**Study**	**Study design**	**Patients (n)**	**% V-A ECMO**	**Outcome studied**	**ICH**		**No ICH**		**RR**	**95% CI**	***p*-value**
					**Non-survivors (*n*)**	**Survivors (*n*)**	**Non-survivors (*n*)**	**Survivors (*n*)**			
Aubron et al. 7	Multicenter, retrospective	149	74	Hospital mortality	5	0	48	96	3.00	2.38–3.78	<0.0001
Davies et al. 10	Multicenter, retrospective	68	7	ICU mortality	6	0	14	48	4.43	2.79–7.02	<0.0001
Fletcher-Sandersjöö et al. 23	Single-center, retrospective	253	36	30 day mortality	44	10	52	134	2.91	2.24–3.79	<0.0001
Kasirajan et al. 14	Single-center, retrospective	74	100	ICU mortality	13	1	36	24	1.55	1.20–1.99	0.001
Klinzing et al. 15	Single-center, retrospective	74	27	6 month mortality	7	1	35	31	1.65	1.17–2.33	0.005
Lockie et al. 16	Single-center, retrospective	250	100	ICU mortality	13	28	44	162	1.48	0.88–2.50	0.137
Luyt et al. 19	Single-center, retrospective	135	0	ICU mortality	7	3	46	81	1.93	1.21–3.08	0.006
Omar et al. 20	Single-center, retrospective	154	81	Hospital mortality	10	2	93	49	1.27	0.96–1.68	0.092
Patroniti et al. 25	Multicenter, retrospective	60	2	ICU mortality	1	0	18	41	3.28	2.23–4.82	<0.0001

ECMO, extracorporeal membrane oxygenation; ICH, intracranial hemorrhage; V-A, venoarterial; RR, risk ratio; CI, confidence interval

While many studies on ICH predictors combined ischemic and hemorrhagic stroke in outcome models (15, 17, 18, 20), four studies analyzed predictors of solely ICH in adult ECMO patients (13, 14, 19, 20). They found that an increased risk of ICH was associated with ECMO-duration (20), pre- and per-ECMO antithrombotic therapy (defined as antithrombotic therapy administered prior to, or during, ECMO treatment) (13, 14), altered intrinsic coagulation (13, 14, 20), renal failure (19), and even pre-ECMO morbidity (13), to name a few. The results are summarized in Table 3. A meta-analysis of ICH predictors was not performed due to the heterogeneity of data.

**Table 3:** Predictors of intracranial hemorrhage in adult patients on extracorporeal membrane oxygenation.

**Risk factor**	**Study**
ECMO duration	Omar et al. 20
Female gender	Kasirajan et al. 14
Pre-admission antithrombotic therapy	Fletcher-Sandersjöö et al. 23
Pre-cannulation SOFA coagulation score	Fletcher-Sandersjöö et al. 23
Thrombocytopenia	Kasirajan et al. 14, Fletcher-Sandersjöö et al. 23
Extracranial bleeding	Omar et al. 20, Fletcher-Sandersjöö et al. 23
Platelet transfusion volume	Omar et al. 20, Fletcher-Sandersjöö et al. 23
RBC transfusion volume	Fletcher-Sandersjöö et al. 23
ACT-levels	Omar et al. 20
Use of heparin	Kasirajan et al. 14
Dialysis	Kasirajan et al. 14
Renal failure at admission	Luyt et al. 19
Hypercreatinemia	Kasirajan et al. 14
PaCO_2_ decrease at ECMO initiation	Luyt et al. 19
PaO_2_ increase at ECMO initiation	Luyt et al. 19

ECMO, extracorporeal membrane oxygenation; SOFA, sepsis organ failure assessment (also known as Severity Organ Failure Assessment); RBC, red blood cell; ACT, activated clotting time; PaCO_2_, partial pressure of carbon dioxide; PaO_2_, partial pressure of oxygen

### Risk of bias

Using the “RTI” item bank (26), bias was assessed in each study that was included in the systematic analysis. Each risk of bias item was graded as either Low-risk or High-risk (Supplementary File 3).

## Discussion

### Summary of main findings

We conducted a systematic review of the incidence, outcome and predictors of ECMO associated ICH in adults. Twenty-five articles were included. We found an ICH-incidence between 1.8 and 21%. Developing an ICH was associated with a mortality of 32–100%, with a relative risk of mortality of 1.27–4.43 in patients that developed ICH as compared to those that did not. To the best of our knowledge, this is the first review of ICH in ECMO-treated adult patients and contributes findings that are important for patient management and future study design.

### Incidence

The reported incidence of ICH in adults during ECMO varied between 1.8 and 21 % (4, 5, 13–22) (Table 1). It is important to note that sedatives and muscle relaxants used during ECMO can mask symptoms of brain injury, resulting in the fact that several ECMO-associated ICH diagnoses are made in the absence of neurological symptoms (15, 23, 27). Moreover, while a CT scan is the gold standard to detect an ICH, in some cases ICH was only detected on magnetic resonance imaging (MRI) (28, 29) or post-mortem autopsies (24, 30). If these tools had been used more frequently, the incidence would presumably have been higher in many studies. Solely relying on neurological assessment before performing a CT scan may, therefore, not be sensitive enough (27, 31). To combat this, some centers perform routine screening cerebral CT scans (13, 16, 31), even though it involves exposure to radiation and potential risks associated with transportation of ECMO patients (32, 33). Centers performing regular screening CT scans had among the highest rate of ICH of the studies included in the analysis (13, 16) (Table 1). Thus, we presume the variation of reported ICH incidence is influenced by centers' routines for performing brain imaging, as well as differences in risk factors between the analyzed cohorts and variability in enrollment criteria.

### Outcome

Survival from ECMO is generally poor, with studies from the Extracorporeal Life Support Organization (ELSO) registry reporting a mortality rate of 38% in V-V ECMO, and 57% in V-A ECMO, patients without neurological complications (17, 18). The systematic review showed a relative risk of mortality of 1.27–4.43 in patients that developed ICH, as compared to those that did not. Only two of the studies showed a non-significant difference (Table 2). Interestingly, one of them was from a center that performed cerebral CT scans at ECMO initiation and adjusted the anti-thrombotic regimen accordingly in those with an ICH present (16). The reasons behind the variance in mortality rates between studies are presumably due to differences in ICH risk factors, variability in enrollment criteria, and variation in clinical thresholds to use CT imaging. For example, a center performing screening CT scans would be more likely to diagnose an ICH with a minor effect on outcome. Unfortunately, the type of ICH and whether it was symptomatic was not described in most of the included studies and therefore not analyzed. The presence of an ICH during ECMO may also result in withdrawal of further therapy (23), thereby introducing a selection bias as it may be considered futile to escalate management in ECMO patients with ICH. Thus, our results indicate that ICH in ECMO patients is generally associated with increased mortality, but that the mortality rates varied between studies.

### Pathophysiology

Alteration in hemostasis is likely a significant mechanism behind ECMO-associated ICH development. ECMO support in and of itself results in thrombocytopenia, factor XIII deficiency, acquired von Willebrand syndrome, fibrinogen deficiency, and pump-induced platelet dysfunction (14, 17, 34–42). Additionally, activation of factor X, and the ensuing production of thrombin, may contribute to further imbalance through consumption of clotting factors (41). All of the above is probably exacerbated by the systemic anticoagulation used to facilitate ECMO (43, 44), and the lack of change in ECMO anticoagulation regimens might be one of the reasons behind the stagnation in rates of ICH occurrence and mortality (17).

Another contributing factor is the systemic inflammatory response that can develop due to activation of circulating blood cells in the ECMO circuit (45), causing simultaneous thrombocytopenia and consumptive coagulopathy (34), as well as the possibility of embolus formation from the cannula that can lead to ischemic stroke in venoarterial (V-A) ECMO and ensuing ICH (27). In a porcine venovenous (V-V) ECMO model, ECMO treatment resulted in increased pro-inflammatory response in cerebral tissue vs. non-ECMO treated animals, highlighting the fact that brain tissue specific inflammation may play a role in the pathophysiology of ICH development (46).

Pre-ECMO factors may also play a role in ICH development. In V-A ECMO, factors related to cardiogenic shock (for example low cerebral blood flow, hypoxia, acidosis, and hemostatic disorders due to liver failure) and reperfusion injury at ECMO initiation can precipitate brain injury (30, 44). In V-V ECMO, abrupt CO_2_, or O_2_ changes at ECMO cannulation can disrupt cerebral perfusion (47, 48), which is further decreased by the use of potent sedatives (49), and has been linked to cerebral desaturation during ECMO initiation (50, 51), as well as impairing cerebral autoregulation which in turn can precede ischemic stroke leading to ICH (19, 52, 53).

Finally, it's noteworthy that given the arbitrary timing of CT-imaging in adult ECMO patients, ICH may result from lesion development before ECMO treatment has even commenced. Supporting this, in one study where cerebral CT scans were performed in all adult ECMO patients at the start of treatment, as many as 16% had an ICH present (16).

Thus, it is likely that ICH etiology is multifactorial, including pre-ECMO morbidity, hemostasis and inflammation.

#### Does V-A ECMO increase the risk of ICH?

It is believed that patients on V-A ECMO are more prone to bleeding complications compared to V-V patients, both due to difference in the underlying clinical condition and comorbidities (7) as well as the ECMO treatment itself. In our systematic review, we could not see any indication that V-A ECMO neither caused an increased risk of ICH, nor that these ICH patients had an increased mortality (Tables 1, 2). However, even though we could not find any association, this is often a matter for debate in the literature. On one hand, it is believed that patients on V-A ECMO are at increased risk of systemic thromboembolism from thrombus formation within the ECMO unit (19), since the blood is returned directly into the arteries without the lungs functioning as a filter. This has been confirmed by studies from the ELSO Registry (17, 18). Moreover, during V-A ECMO cerebral perfusion is mainly non-pulsatile (unless combined with an intra-aortic balloon pump) (54), although it is unclear whether this affects the risk of ICH. In addition to differences in ECMO treatment itself, there is a difference in the underlying clinical condition and comorbidities (7). Despite this, one study from the ELSO Registry found that ICH was twice as likely to occur in V-V ECMO patients compared to V-A (17, 18), and single-center studies of V-A and V-V ECMO adult patients have not identified ECMO-mode as a predictor of ICH development (13, 20). Thus, additional research is needed to determine if V-A ECMO really does increase the risk of ICH in adult ECMO patients.

### Management

Treating an ICH during ECMO represents a difficult balance between pro- and anticoagulatory demands. Hematoma components, patient characteristics, and other predictors of outcome need to be assessed to determine the best course of action (23). Only one single-center retrospective cohort study has described the management of ICH in adults during ECMO support (23). In this study, a decision was made to withdraw life-sustaining ECMO therapy in 42% of the ICH patients, no intervention was undertaken in 18% because the ICH was deemed to be of minimal clinical importance, and 40% of patients had medical and/or surgical treatment. Interestingly, in patients where no intervention was done there were no deaths attributed to the ICH, presumably due to smaller hematoma volumes and/or non-critical ICH location (23).

While surgical management may be indicated when an ICH occurs during ECMO, the associated anticoagulation presents a considerable risk. Moreover, time restraints allow limited opportunity for pre-operative optimization of coagulation, other than immediate heparin reversal, to decrease intra- and postoperative blood loss and hematoma progression. We have identified nine cases of surgical intervention in adult patients with ECMO-associated ICH (23, 55–58), with two survivors (23, 57). Thus, neurosurgical intervention in patients with ongoing anticoagulation is extremely hazardous, but the two successful case reports suggest that it might be an option in well-selected patients where no other management strategies are available.

### Future perspectives

Given the increasing utilization of ECMO and the poor outcomes associated with ICH, more research is needed to determine the best way to prevent ICH from occurring and/or progressing. A better understanding of the pathophysiology and predictors of ECMO-associated ICH will facilitate identification of patients who are more prone to developing the complication, and where more rigorous neurological checks, earlier weaning from ECMO, or alternatives for anticoagulation could be considered.

#### Hemostasis

There have been several successful case reports of V-V and V-A ECMO treatment without systemic anticoagulation in hemorrhagic patients (59–61), as well as on patients requiring ECMO after traumatic brain injury (62–65). Moreover, a study comparing anticoagulation guided with activated clotting time (ACT) levels of 180–220 s (s) vs. 140–160 s showed no difference in oxygenator failure caused by clotting (66). In another study of adult V-V ECMO patients, prophylactic correction of coagulation factor deficiencies led to a reduction in the rate of ICH (34). In rabbits, recombinant fully human antibody 3F7, which interferes with activated factor XII (FXIIa) mediated coagulation, provided thrombo-protection as efficiently as heparin but did not impair the hemostatic capacity or increase bleeding from wounds, suggesting that FXIIa targeting could work as a anticoagulation strategy not associated with excess bleeding (67). Another V-V ECMO study evaluated a protocol where 61 patients were assigned subcutaneous enoxaparin exclusively, and no cases of ICH or oxygenator change due to clotting were observed (68). Lastly, the pilot study for the HELP-ECMO study, evaluating normal vs. low-dose heparin in adult patients, resulted in significant decreases in daily activated partial thromboplastin time and anti-Xa levels but no difference in thromboembolic or bleeding events, thus supporting the feasibility of a phase III study evaluating the effectiveness of low-dose anticoagulation in adult patients during ECMO (69).

Considering the fact that ECMO causes platelet dysfunction (42), which can lead to the development of ICH even in the absence of thrombocytopenia (70), one should also consider the value of performing regular platelet function tests (such as platelet aggregometry, Multiplate®) during treatment, which has revealed different temporal trajectories of platelet receptor activity following traumatic brain injury (71). Tentative evidence from smaller studies indicate that multiplate values during ECMO may facilitate in the detection of patients at risk of bleeding events (72, 73).

#### Non-invasive neurological monitoring

There is a difficulty associated with neurological assessment of ECMO patients, and the fact that invasive monitoring procedures are associated with a high risk of uncontrolled bleeding and death (23), non-invasive neurological monitoring could provide a suitable option for these patients in order to detect ICH development and initiate eventual treatment efforts at an earlier stage. This includes, but is not limited to, protein biomarkers of brain injury (74), cerebral near infrared spectroscopy (NIRS) (75), and transcranial doppler (TCD) to assess dynamics of intracerebral vessels and to assess the intracranial pressure (ICP) (76).

S100B, a biomarker used to monitor treatment effect and detect secondary brain damage (77–79), or rule out traumatic ICH after moderate-to-mild TBI (80), has been assessed during ECMO in a few smaller studies. A case-control study showed increased S100B levels in ECMO-treated infants with ICH up to 72 h before they were detectable on a cranial ultrasound (81). Another case-control study of 15 ECMO-treated patients found that S100B was significantly higher in the group with cerebral complications (82). Bembea et al. found that the biomarkers S100B, neuron specific enolase (NSE), glial fibrillary acidic protein (GFAP), and monocyte chemoattractant protein 1 (MCP1) levels were higher in non-survivors and in patients with unfavorable outcome in neonatal and pediatric ECMO cohorts (83). Another study showed that GFAP was elevated 1–2 days before neurological injury was diagnosed using neuroimaging in pediatric ECMO patients (84). High NSE levels after extracorporeal cardiopulmonary resuscitation have also been shown to correspond to poor neurological outcome and mortality (85). Thus, there is tentative evidence of biomarkers' utility to detect ICH during ECMO treatment, but larger trials investigating their clinical usefulness as part of a decision algorithm to perform a head CT or other imaging to detect ECMO-associated ICH in adults are warranted.

NIRS uses near-infrared light to measure trends in cerebral oxygenation (86). In neonatal ECMO patients, case series have reported decreased brain tissue oxygenation in patients that later demonstrated cerebral injury on neuroimaging (87). Only one study has studied cerebral NIRS in adults, finding that cerebral oxygenation significantly responded to hemodynamic intervention and that non-responders were more likely to have ischemic cerebral complications (88).

We have not found any studies that have evaluated the use of TCD for detection of ICH in adult ECMO patients. However, in a pediatric ECMO study TCD detected abnormally high blood flow velocities several days prior to detection of an ICH (89), thus warranting further studies into the clinical utility of TCD to predict ICH in ECMO patients.

## Limitations

There are some limitations to this study that should be mentioned. First and foremost, ECMO cohorts carry an inherent heterogeneity due to factors such as variations in centers' patient acceptance criteria and disease panorama. Moreover, due to a lack of data we were not able to analyze several established predictors of ICH mortality, such as hematoma volume, hematoma location, and secondary complications (90–92). We also included several forms of ICH (i.e., IPH, SDH, and SAH) in common analysis. Thus, we were not able to perform more extensive systematic analysis, such as meta-analysis of mortality rates or risk factors for ICH development.

## Conclusion

ICH is a common complication in adults treated with ECMO and associated with increased mortality. Evidence shows that it is to some extent caused by both pre-ECMO morbidity and ECMO-induced disruption of hemostasis. Treating an ICH during ECMO represents a complicated balance between pro- and anticoagulatory demands. Neurosurgical treatment is associated with severe morbidity, but has been successful in selected cases. A better understanding of the pathophysiology and predictors of ECMO-associated ICH may help reduce its incidence. Moreover, prospective trials are warranted to investigate the validity and feasibility of non-invasive neuromonitoring in early detection of the complication.

## Author contributions

AF-S, ET, and JB study design. AF-S data collection. AF-S and ET statistical analysis. All authors data interpretation, revision and approval of manuscript. AF-S draft of manuscript. BB study supervision.

### Conflict of interest statement

The authors declare that the research was conducted in the absence of any commercial or financial relationships that could be construed as a potential conflict of interest. The reviewers SP and NO and handling Editor declared their shared affiliation.

## Abbreviations

ACT: Activated clotting time
CT: Computed tomography
ECMO: Extracorporeal membrane oxygenation
ELSO: Extracorporeal Life Support Organization
FXIIa: Activated factor XII
GFAP: Glial fibrillary acidic protein
ICP: Intracranial pressure
IPH: Intraparenchymal pressure
MCP1: Monocyte chemoattractant protein 1
MRI: Magnetic resonance imaging
NIRS: Near infrared spectroscopy
NSE: Neuron specific enolase
PRISMA: Preferred Reporting Items for Systematic Reviews and Meta-
SAH: Subarachnoid hemorrhage
SDH: Subdural hemorrhage
TCD: Transcranial doppler
V-A: Venoarterial
V-V: Venovenous.
