# Supplementary material for: Incidence, Outcome, and Predictors of Intracranial Hemorrhage in Adult Patients on Extracorporeal Membrane Oxygenation: A Systematic and Narrative Review
Source: Front Neurol. 2018 Jul 6;9:548. doi: 10.3389/fneur.2018.00548 (PMC6043665; doi:10.3389/fneur.2018.00548)
Supplement: Supplementary file 1 [file Table_1.DOCX]

**MEDLINE and EMBASE Search Strategy**

1. extracorporeal membrane oxygenation.mp. [mp=ti, ab, hw, tn, ot, dm, mf, dv, kw, fs, bt, id, cc, nm, kf, px, rx, an, eu, pm, ui]
2. extracorporeal membrane oxygenation.tw.
3. ECMO.mp.
4. ECMO.tw.
5. extracorporeal life support.mp.
6. extracorporeal life support.tw.
7. ECLS.mp.
8. ECLS.tw.
9. 1 or 2 or 3 or 4 or 5 or 6 or 7 or 8
10. intracranial hemorrhage.mp.
11. intracranial hemorrhage.tw.
12. intracerebral hemorrhage.mp.
13. intracerebral hemorrhage.tw.
14. brain hemorrhage.mp.
15. brain hemorrhage.tw.
16. brain injury.mp.
17. brain injury.tw.
18. neurological injury.mp.
19. neurological injury.tw.
20. neurological complications.mp.
21. neurological complications.tw.
22. 10 or 11 or 12 or 13 or 14 or 15 or 16 or 17 or 18 or 19 or 20 or 21

Search string was “9 and 22”
