# Supplementary material for: Incidence, Outcome, and Predictors of Intracranial Hemorrhage in Adult Patients on Extracorporeal Membrane Oxygenation: A Systematic and Narrative Review
Source: Front Neurol. 2018 Jul 6;9:548. doi: 10.3389/fneur.2018.00548 (PMC6043665; doi:10.3389/fneur.2018.00548)
Supplement: Supplementary file 3 [file Table_3.DOC]

Bias table of studies that reported mortality in both ICH and non-ICH adult ECMO cohorts

| **Reference** | **Inclusion/Exclusion Criteria** | **Comparison Group** | **Recruiting** | **Blinding** | **Valid Measures of Outcome** | **Follow Up Length** | **Attrition Assessment** | **Selective Outcome Reporting** | **Overall Bias Assessment** |
| --- | --- | --- | --- | --- | --- | --- | --- | --- | --- |
| Aubron 2016 | L | L | H | H | H | L | L | L | L |
| Davies 2009 | L | L | H | H | H | L | L | L | L |
| Fletcher-Sandersjöö 2017 | L | L | H | H | H | L | L | L | L |
| Kasirajan 1999 | L | L | H | H | H | L | L | L | L |
| Klinzing 2017 | L | L | H | H | H | L | L | L | L |
| Lockie 2017 | L | L | H | H | H | L | L | L | L |
| Luyt 2016 | L | L | H | H | H | L | L | L | L |
| Omar 2016 | L | L | H | H | H | L | L | L | L |
| Patroniti 2011 | L | L | H | H | H | L | L | L | L |

Abbreviations: ICH= intracranial hemorrhage; ECMO= extracorporeal membrane oxygenation; H= high risk of bias; L= low risk of bias

**Factors Considered in the Bias Assessment**

1. Inclusion/Exclusion criteria – studies were assessed on the extent of their listed criteria, and if the criteria varied across the comparison groups.
2. Comparison Group – studies were assessed on the strategy for recruiting participants and if the method differed across groups.
3. Recruiting – retrospective database mining was considered high risk. Poorly described methods of patient recruitment/consent were considered biased. Small sample size was considered a form of bias.
4. Blinding – lack of blinding of data to outcome measure assessment was considered a source of bias.
5. Valid Outcome Measures – were valid and reliable measures not used or not implemented consistently across all study participants to assess inclusion/exclusion criteria, intervention/exposure outcomes, participant benefits and harms, and potential confounders?
6. Duration of Follow-up – studies were considered high-bias if duration of follow-up was not defined, different across study groups or poorly documented.
7. Attrition – missing data were considered for potential bias in the consideration of attrition.
8. Selective Outcome Reporting – were there any important primary outcomes missing from the results?
9. Overall Assessment – summative decision if the results were believable taking study limitations into consideration.

**Summary**

Both single- and multicenter studies were included in the systematic review. All studies included patient treated with ECMO within a certain time period. A negligible amount of patients were lost to follow-up. Overall, the studies were deemed low-risk for bias. There was no variation in individual categories, and high-risk biases were associated with recruiting, lack of blinding and risk for confounders.
